# The Pivotal Role of a Novel Biomarker of Reactive Oxygen Species in Chronic Kidney Disease

**DOI:** 10.1097/MD.0000000000001040

**Published:** 2015-06-26

**Authors:** Yoshihiro Hirata, Eiichiro Yamamoto, Takanori Tokitsu, Koichiro Fujisue, Hirofumi Kurokawa, Koichi Sugamura, Kenji Sakamoto, Kenichi Tsujita, Tomoko Tanaka, Koichi Kaikita, Seiji Hokimoto, Seigo Sugiyama, Hisao Ogawa

**Affiliations:** From the Department of Cardiovascular Medicine, Faculty of Life Sciences, Graduate School of Medical Science, Kumamoto University, Kumamoto, Japan.

## Abstract

Risk stratification of chronic kidney disease (CKD) is clinically important because such patients are at high risk of cardiovascular events. Although reactive oxygen species (ROS) are reported to be closely associated with the pathophysiology of CKD, there are few useful ROS biomarkers known for CKD patients. Hence, our objectives in this study were to investigate whether serum derivatives of reactive oxygen metabolites (DROM), a novel biomarker of ROS, is involved in the pathophysiology of CKD (case-control study), and is a significant predictor of future cardiovascular events in CKD patients (follow-up study).

Patients with suspected coronary artery disease (CAD) were enrolled and underwent coronary angiography. Patients with CKD (estimated glomerular filtration ratio <60 mL/min/1.73 m^2^ and/or proteinuria, *n* = 324) were compared with those without CKD (non-CKD). Serum DROM was measured at stable conditions. A case-control study of the 324 CKD patients and 263 non-CKD patients was conducted after matching risk factors, and a follow-up study of the 324 CKD patients was performed. CKD patients were divided into low- and high-DROM groups using their median value (348 unit; called the Carratelli unit [U.CARR]), and followed until the occurrence of cardiovascular events.

DROM levels were significantly higher in risk factors-matched CKD patients than in risk factors-matched non-CKD patients (347.0 [301.8–391.8] U.CARR vs. 338.5 [299.8–384.3] U.CARR, *P* = 0.03). During mean 23 ± 14 months follow-up of 324 CKD patients, 83 cardiovascular events were recorded. Kaplan–Meier analysis demonstrated a higher probability of cardiovascular events in CKD patients with high DROM than in those with low DROM (*P* < 0.001, log-rank test). Multivariate Cox hazard analysis including significant predictors in simple Cox hazard analysis demonstrated that high DROM was a significant and independent predictor of cardiovascular events in CKD patients (hazard ratio: 1.76, 95% confidence interval: 1.10–2.82, *P* = 0.02).

In conclusion, serum DROM values were significant and independent predictors of cardiovascular events in CKD patients, indicating that the measurements of DROM might provide clinical benefits for risk stratification of CKD patients.

## INTRODUCTION

The number of patients with chronic kidney disease (CKD) has been increasing progressively worldwide. Because coronary artery disease (CAD) is the major cause of death among cardiovascular events in CKD patients,^1^ cardiovascular risk stratification of CKD patients is clinically important. Coronary angiography (CAG) is one of the most useful methods to evaluate the presence and severity of CAD; however, CKD patients are known to be predisposed to contrast-induced nephropathy.^2^ Therefore, the use of invasive methods such as CAG in CKD patients is undesirable, and noninvasive methods to evaluate cardiovascular risk are needed.

Renal dysfunction can develop in various diseases, such as hypertension, diabetes mellitus (DM), glomerular diseases, and collagen diseases, and reactive oxygen species (ROS) are known to be widely associated with the development of CKD in these diseases. Although recent clinical reports have shown positive associations between CKD and ROS,^3^ there are few reports identifying a significant association between ROS and cardiovascular events in CKD patients.

There are numerous methods to evaluate oxidative status in vivo using blood or urine samples; however, few ROS biomarkers have been identified for CKD patients because of its unstable character and poor reproducibility. The hydroperoxide level, a major ROS, measured using the ferrous oxidation of xylenol orange 2 assay, is reported to be a useful prognostic factor in CAD patients.^4^ We recently reported the feasibility of serum derivatives of reactive oxygen metabolites (DROM), which also reflect hydroperoxide levels, in risk stratification of CAD patients.^5^ In the present study, we investigated whether DROM is a useful ROS biomarker in patients with CKD.

## METHODS

### Study Participants and Protocol

**FIGURE 1:**
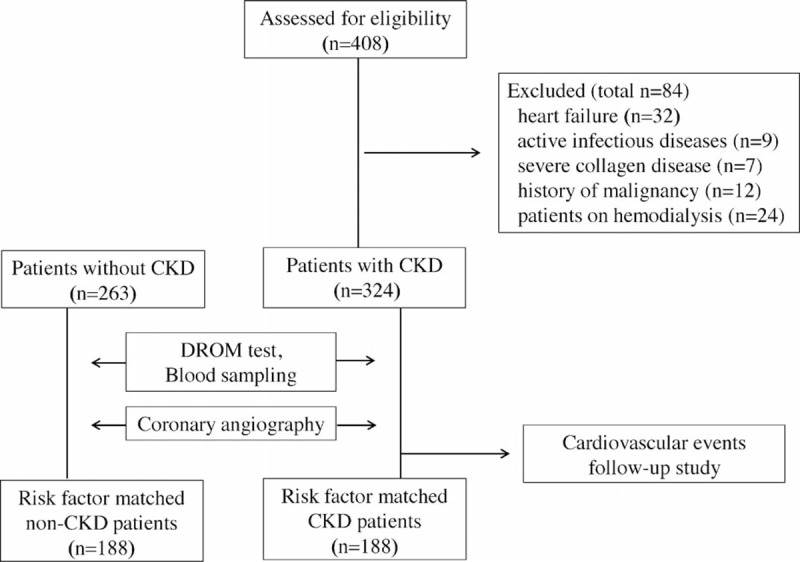
Flow chart showing the study protocol.

We initially recruited 408 consecutive patients with CKD who were hospitalized at Kumamoto University Hospital for CAG because of suspected CAD between January 2007 and June 2014. Eighty-four patients were excluded: heart failure (n = 32), active infectious diseases (n = 9), severe collagen disease (n = 7), history of malignancy (n = 12), and end-stage renal dysfunction patients on hemodialysis (n = 24). Finally, 324 CKD patients were enrolled (Figure 1).

First, a case-control study was performed to evaluate oxidative status in CKD patients. Serum DROM levels were measured and compared between patients with CKD (n = 324) and those without CKD (non-CKD) (n = 263). To investigate whether DROM levels increased with only CKD influence, DROM levels were further compared between risk factor-matched non-CKD patients (n = 188) and risk factor-matched CKD patients (n = 188) after matching risk factors (number of patients, age, sex, equal incidence of CAD, hypertension, DM, and dyslipidemia) using propensity score matching.

Second, CKD patients were divided into 2 groups using median value of DROM (348 unit; called the Carratelli unit [U.CARR]); high-DROM group (n = 164) and low-DROM group (n = 160). A follow-up study was conducted for these 324 CKD patients. They were followed until August 2014 or the occurrence of cardiovascular events.

The study protocol conformed to the principles of the Declaration of Helsinki. Written informed consent was obtained from all patients. The study is registered at the University Hospital Medical Information Network Clinical Trials Registry (UMIN000015474).

### Definition of CKD

CKD was defined as low if the estimated glomerular filtration ratio (eGFR) <60 mL/min/1.73 m^2^ or high if the eGFR >60 mL/min/1.73 m^2^ with proteinuria. eGFR was calculated using the Japanese Society of Nephrology formula.^6^ Urinary protein was evaluated semiquantitatively using a urine dipstick test (Uro-Labstix; Siemens Japan, Tokyo, Japan). Proteinuria was defined as urinary protein excretion >30 mg/dL.

### Definition of Coronary Risk Factors

DM was defined as symptoms of DM and a casual plasma glucose concentration ≥200 mg/dL, fasting plasma glucose concentration ≥126 mg/dL, 2-h plasma glucose concentration ≥200 mg/dL from a 75-g oral glucose tolerance test, or taking medication for DM. Hypertension was defined as >140/90 mm Hg or taking antihypertensive medication. Current smoking was defined as smoking at admission. Dyslipidemia was defined as high-density lipoprotein cholesterol <40 mg/dL or low-density lipoprotein cholesterol >140 mg/dL, triglycerides >150 mg/dL or taking medication for dyslipidemia.

### Estimation of the Presence and Severity of CAD

All CKD patients underwent CAG. CAD was defined as coronary artery stenosis greater than 75% narrowing of the arterial diameter in at least 1 coronary artery using quantitative CAG. CAD severity was determined using Gensini scores, which are reported to be associated with various coronary risk factors.^7^ Gensini scores were calculated using the Gensini coronary artery scoring method (Gensini score = stenosis score × importance of arterial function score).^8^

### Blood Sampling

Blood tests were performed early in the morning at our hospital laboratory after patients had fasted. Serum creatinine, calcium, phosphorus, high-sensitivity C-reactive protein (hs-CRP), B-type natriuretic peptide (BNP), blood glucose, hemoglobin A1c, total cholesterol, high-density lipoprotein cholesterol, low-density lipoprotein cholesterol, and triglyceride levels were measured.

### DROM Test

The oxidative status of CKD patients was measured using the DROM test (Diacron International srl, Grosseto, Italy). The principles and reproducibility of the DROM test have been described previously.^9,10^ Serum DROM was measured under stable conditions. Serum DROM levels reflect total oxidant capacity, comprising hydroperoxide, ferroxidase activity, and myeloperoxidase activity. Normal reference levels of DROM given by the manufacturer (Diacron) are 250–300 U.CARR.^9,10^

### Follow-Up Study and Definitions of Cardiovascular Events

The 324 CKD patients were followed until August 2014 or until the occurrence of cardiovascular events after assessment of DROM levels. Cardiovascular events were identified from medical records, and were confirmed by direct contact with patients, their families or physicians. Definitions of cardiovascular events were as follows: cardiovascular death, nonfatal myocardial infarction, unstable angina pectoris, nonfatal ischemic stroke, hospitalization for heart failure decompensation, and coronary revascularization. Cardiovascular death was defined as death due to cardiovascular diseases (myocardial infarction, heart failure, documented sudden death in the absence of noncardiovascular causes). Myocardial infarction was diagnosed by the rise or fall of cardiac biomarkers (plasma creatine kinase-MB, troponin T) above the 99th percentile of the upper limit of the normal range and evidence of myocardial ischemia (ischemic changes on electrocardiogram or imaging evidence of new loss of viable myocardium or new abnormality of regional wall motion). Unstable angina pectoris was diagnosed by new or accelerating symptoms of myocardial ischemia with new ischemic changes on electrocardiogram. Ischemic stroke was diagnosed by focal neurological deficits with radiological evidence of brain infraction excluding intracranial hemorrhage. Heart failure decompensation was diagnosed by hospitalization with symptoms typical of heart failure and objective signs of worsening heart failure requiring intravenous drug administration. Coronary revascularization was diagnosed by performed percutaneous coronary intervention or coronary artery bypass grafting because of myocardial ischemia.

### Statistical Analysis

The Kolmogorov–Smirnov test was used to assess normal distribution of continuous data. Nonnormally distributed data are expressed as median (25–75%). Continuous variables with normal distribution are expressed as mean ± standard deviation. Categorical data are presented as frequencies and percentages. Differences between 2 groups were tested using the Fisher exact test for categorical variables. Differences in continuous variables were analyzed using the unpaired t-test or Mann–Whitney *U* test, as appropriate. Propensity score matching methods were used to match risk factors between CKD and non-CKD patients. Independent variables included in the propensity score model were age, sex, proportion of CAD, hypertension, DM, and dyslipidemia. Kaplan–Meier analysis was performed using the median value of DROM (348 U.CARR) in CKD patients, and cardiovascular event incidence was compared using the log-rank test. The Cox proportional hazard model was used to estimate the cardiovascular event hazard ratio (HR) and its 95% confidence interval (CI) in CKD patients by simple and multivariate analyses with forced inclusion models. Significant clinical parameters associated with cardiovascular events in simple Cox hazard analysis were entered into multivariate Cox hazard analysis. Nonnormally distributed data were transformed into natural logarithmic values in Cox hazard analysis and correlation analysis. A *P*-value <0.05 was considered statistically significant. Statistical analyses were performed using The Statistical Package for Social Sciences version 20 (SPSS Inc, Tokyo, Japan).

## RESULTS

### Serum DROM Levels in Non-CKD and CKD Patients

Risk factor-matched non-CKD patients (n = 188) were compared with risk factor-matched CKD patients (n = 188). Baseline characteristics are shown in Table 1. DROM levels were significantly higher in risk factor-matched CKD patients than in risk factor-matched non-CKD patients (347.0 [301.8–391.8] U.CARR vs. 338.5 [299.8–384.3] U.CARR, *P* = 0.03). Plasma BNP and serum hs-CRP levels were significantly higher (*P* < 0.001 and *P* = 0.04), and eGFR was significantly lower (*P* < 0.001) in risk factor-matched CKD patients than in risk factor-matched non-CKD patients. The proportions of use of β-blockers, renin angiotensin system blockers (angiotensin-converting enzyme inhibitors [ACE-I] and/or angiotensin II receptor blockers [ARB]), and loop diuretics were significantly higher in risk factor-matched CKD patients than in risk factor-matched non-CKD patients (*P* = 0.03, *P* = 0.02, *P* < 0.001, respectively, Table 1).

**TABLE 1:**
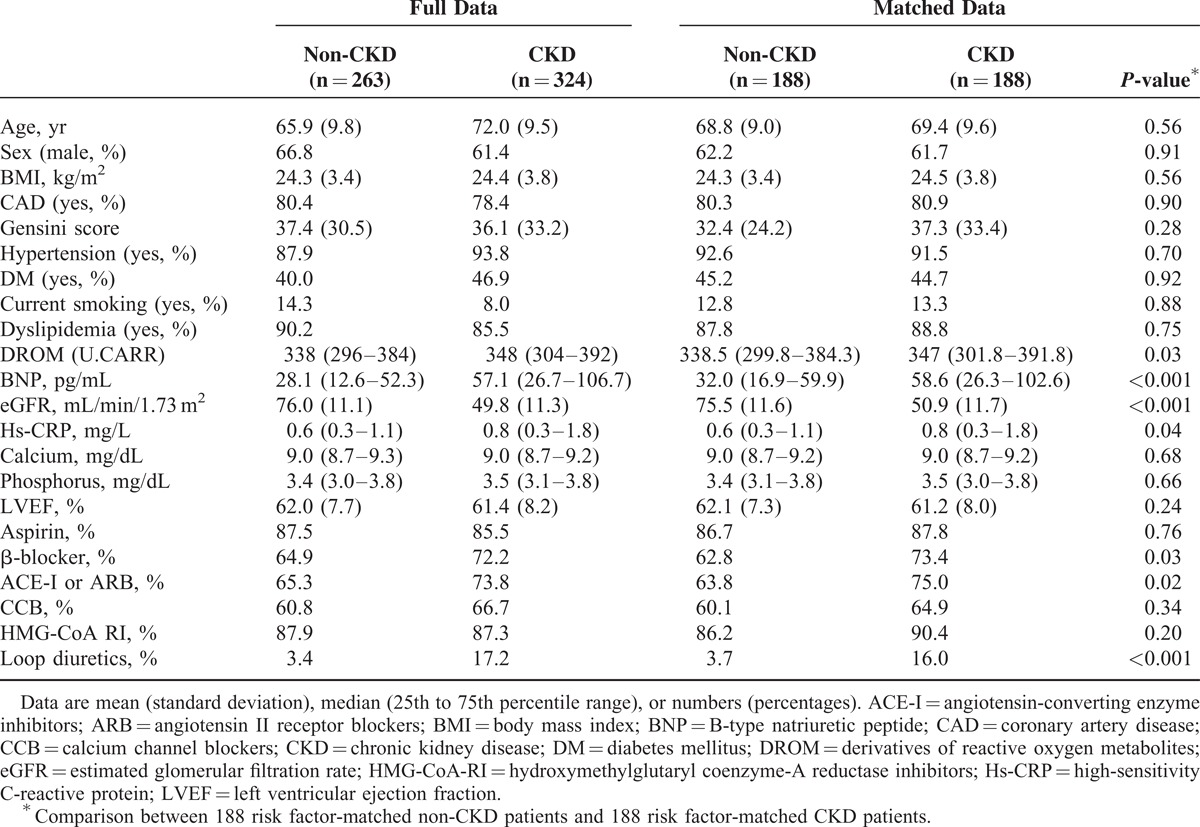
Baseline Characteristics of All CKD Patients, All Non-CKD Patients, 188 Risk Factor-Matched Non-CKD Patients, and 188 Risk Factor-Matched CKD Patients

### Baseline Characteristics of 324 CKD Patients

The 324 CKD patients were divided into a low-DROM group (n = 160) and a high-DROM group (n = 164) using the median value of DROM (348 U.CARR). CKD patients with high-DROM were mainly women (*P* < 0.001), and had higher body mass index, BNP, hs-CRP, and phosphorus levels (*P* = 0.04, *P* = 0.03, *P* = 0.03, *P* = 0.008, respectively). The prevalence of CAD and Gensini score were significantly higher in CKD patients with high-DROM than in those with low-DROM (*P* = 0.02 and *P* = 0.03). The proportions of use of renin angiotensin system blockers (ACE-I and/or ARB) and loop diuretics were significantly higher in CKD patients with high-DROM than in those with low-DROM (*P* < 0.001 and *P* = 0.01, Table 2).

**TABLE 2:**
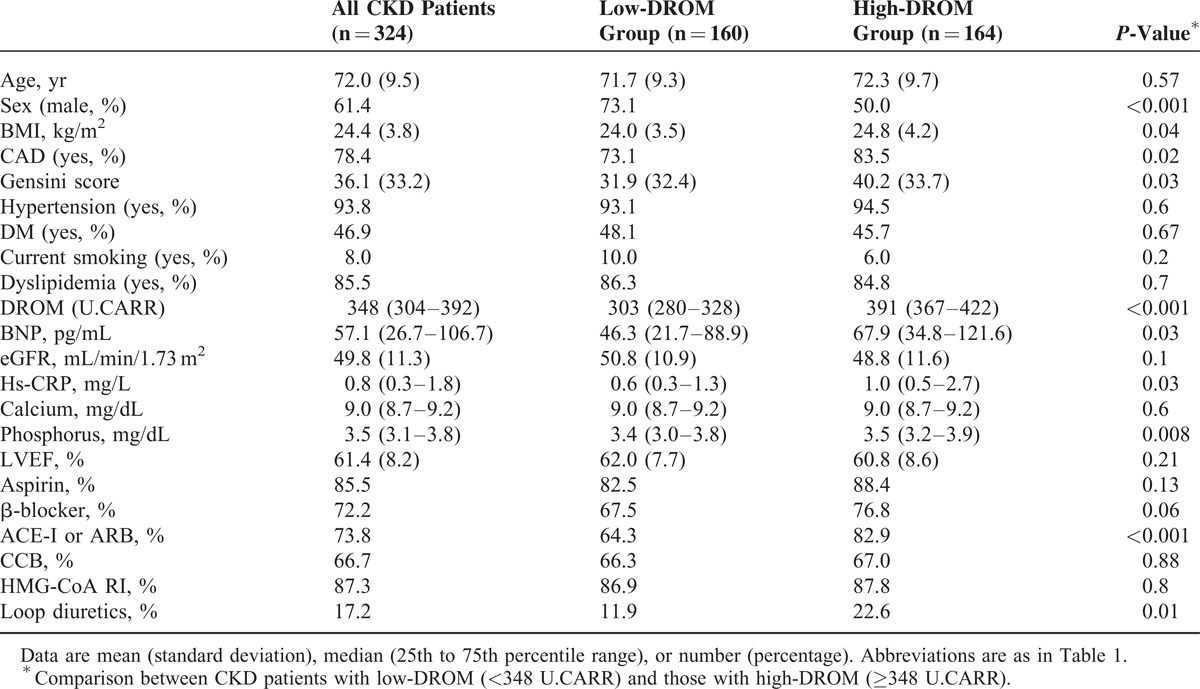
Baseline Characteristics of 324 CKD Patients

### Association of DROM With Other Biomarkers

**FIGURE 2:**
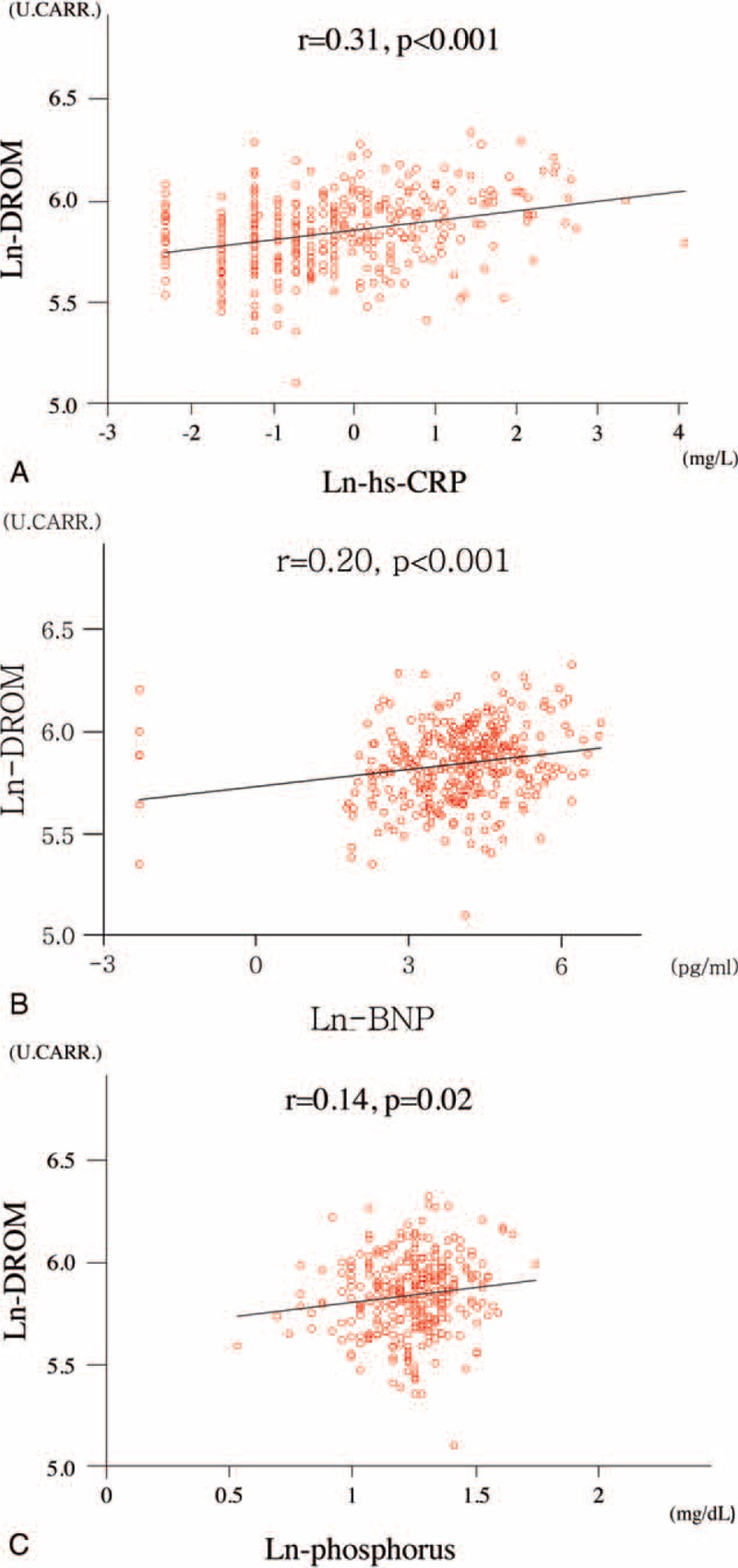
Correlations between ln-DROM and other biomarkers. A, Correlation between ln-DROM and ln-hs-CRP. B, Correlation between ln-DROM and ln-BNP. C, Correlation between ln-DROM and ln-phosphorus. *r*: correlation coefficient. Because serum DROM, hs-CRP, phosphorus, and plasma BNP levels were not normally distributed, we calculated the natural logarithmic transformed values as ln-DROM, ln-hs-CRP, ln-phosphorus, ln-BNP for analysis.

The correlation between DROM and other clinical biomarkers was investigated. Because serum DROM, hs-CRP, phosphorus and plasma BNP levels, and eGFR were not normally distributed, we calculated the natural logarithmic transformed values as ln-DROM, ln-hs-CRP, ln-phosphorus, ln-BNP, and ln-eGFR. There was no significant correlation between ln-DROM and ln-eGFR levels (data not shown). There were significant and positive correlations of ln-DROM with ln-hs-CRP (correlation coefficient: *r* = 0.31, *P* < 0.001), ln-BNP (*r* = 0.20, *P* < 0.001), and ln-phosphorus (*r* = 0.14, *P* = 0.02) (Figure 2A–C).

### Presence of CAD in 324 CKD Patients

All CKD patients underwent CAG to evaluate the presence and severity of CAD. There were 70 patients without CAD (non-CAD) and 254 patients with CAD. Baseline characteristics are shown in Table 3. CKD patients with CAD were significantly older and had a higher Gensini score than with non-CAD (both *P* < 0.001). The proportions of CKD patients with high-DROM, DM, and dyslipidemia were significantly higher (*P* = 0.02, *P* = 0.03, *P* = 0.001, respectively). The proportions of use of aspirin, β-blockers, and hydroxymethylglutaryl coenzyme-A reductase inhibitors were significantly higher in CKD patients with CAD than in those with non-CAD (all *P* < 0.001, Table 3).

**TABLE 3:**
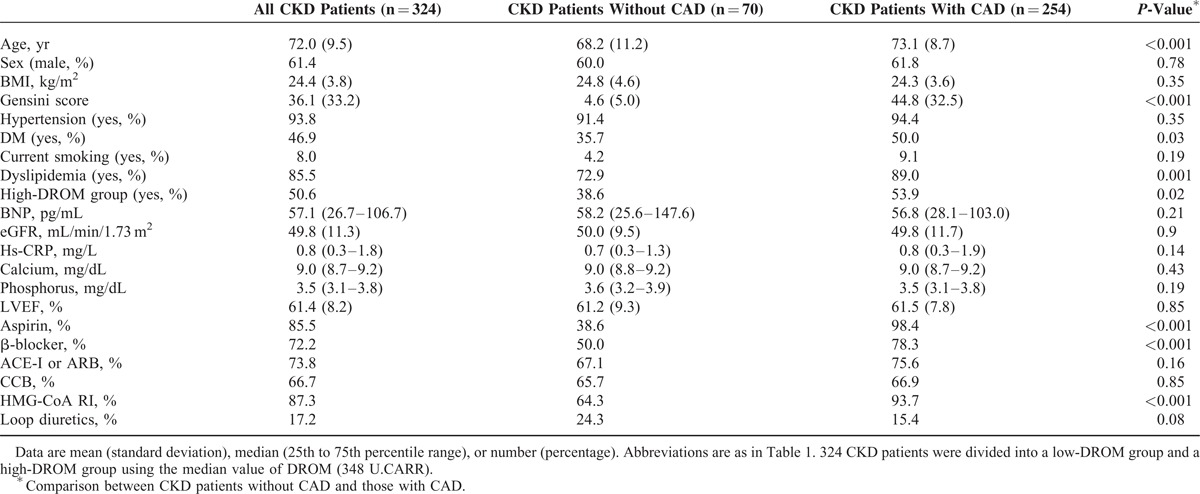
Baseline Characteristics of 324 CKD Patients With or Without CAD

### Follow-Up Study of 324 CKD Patients

**FIGURE 3:**
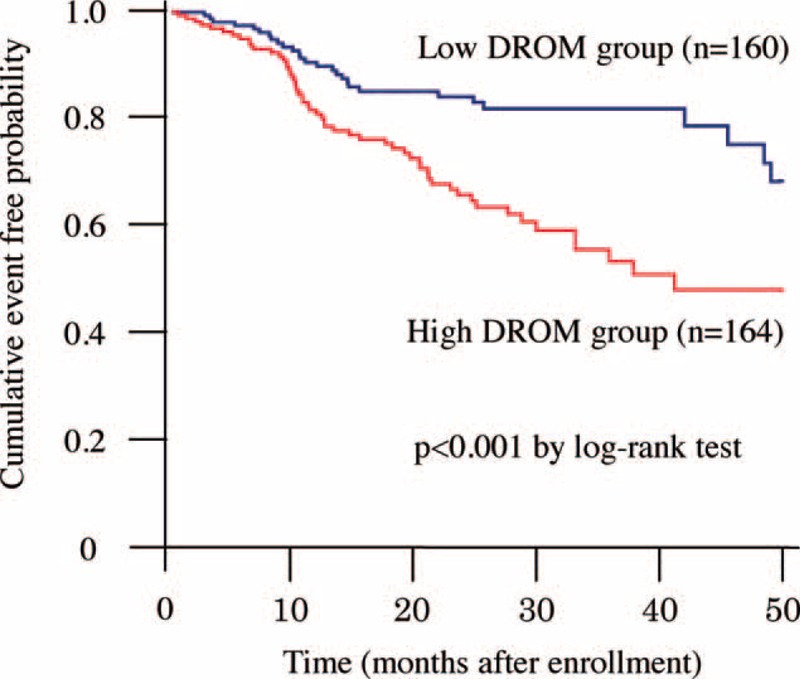
Follow-up analysis. Kaplan–Meier analysis for the probability of cardiovascular events in CKD patients from low- and high-DROM groups after CKD patients were divided into 2 groups using the median value of DROM (348 U.CARR). CKD patients with high-DROM had a significantly higher probability of cardiovascular events compared with those with low-DROM.

CKD patients were followed until the occurrence of cardiovascular events or August 2014. During mean 23 ± 14 months follow-up, 83 cardiovascular events were recorded. Details of cardiovascular events are shown in Table 4. Kaplan–Meier analysis demonstrated a significantly higher probability of cardiovascular events in CKD patients with high-DROM compared with low-DROM (cut-off value of DROM: 348 U.CARR; log-rank test, *P* = 0.01; Figure 3).

**TABLE 4:**
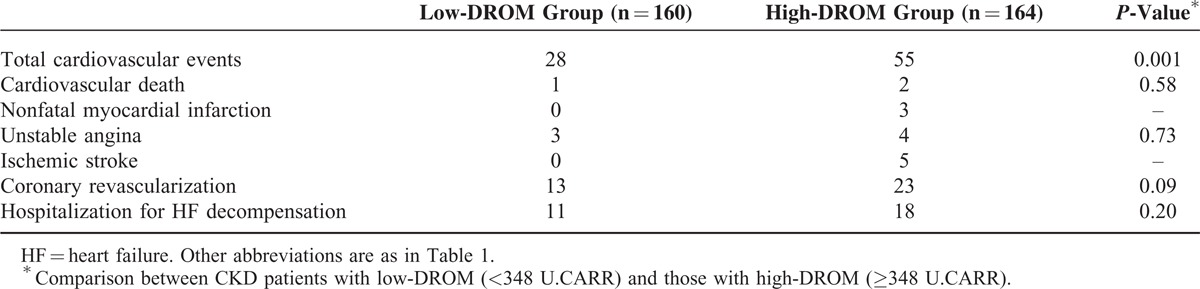
The Occurrence of Cardiovascular Events in CKD Patients From Low- and High-DROM Groups

Univariate cox proportional hazard analysis for cardiovascular events identified 5 variables as significant predictors (presence of CAD, high-DROM, serum ln-calcium level, plasma ln-BNP level, and ln-left ventricular ejection fraction). Multivariate cox proportional hazard analysis, including significant predictors in univariate cox proportional hazard analysis, identified that high-DROM was a significant and independent predictor for future cardiovascular events in CKD patients (HR: 1.76, 95% CI: 1.10–2.82, *P* = 0.02, Table 5).

**TABLE 5:**
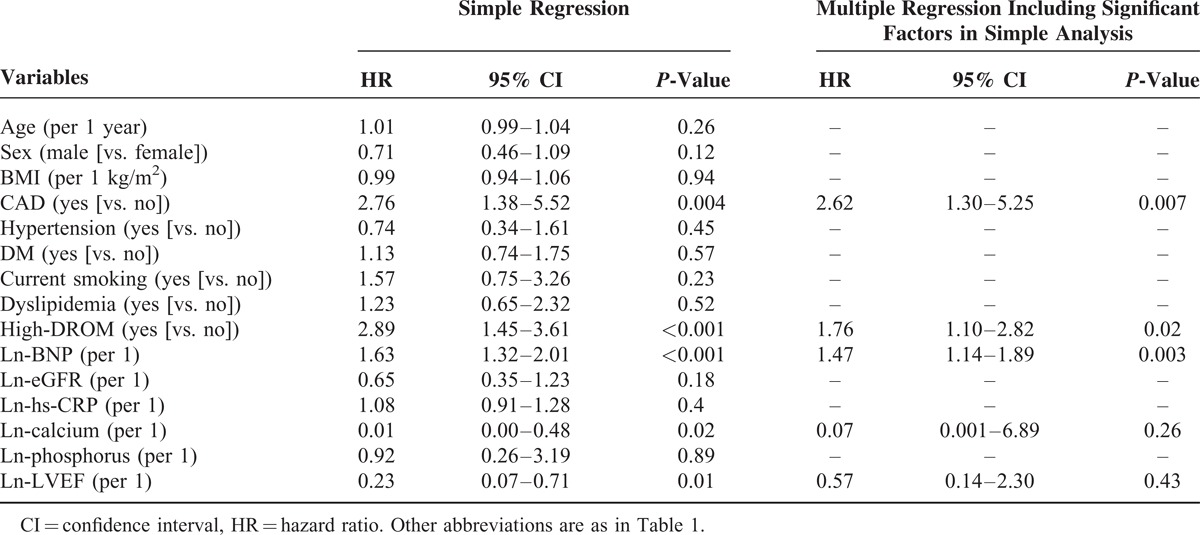
Cox Proportional Hazards Analysis for Future Cardiovascular Events in CKD Patients

## DISCUSSION

This study identified the following: serum DROM levels were significantly higher in CKD patients than in risk factor-matched non-CKD patients; CKD patients with CAD had higher DROM levels than those without CAD; Kaplan–Meier analysis showed a significantly higher probability of cardiovascular events in CKD patients with high-DROM compared with low-DROM; and multivariate Cox hazard analysis identified high-DROM as an independent and significant predictor of future cardiovascular events in CKD patients.

Using Dahl salt-sensitive hypertensive rats, a useful model of not only hypertension but also vascular injury, including atherosclerosis, we previously found that ROS are closely associated with the pathogenesis of atherosclerosis and CKD.^11,12^ Coronary atherosclerosis develops into obstructive CAD, and previous clinical studies have reported that ROS were involved in the occurrence and development of CAD.^4,13^ However, direct measurements of ROS are difficult because of their biochemical instability, and few biomarkers of ROS have been adopted for clinical examination.

The DROM test is a simple, novel, and relatively inexpensive integrated analytical system to measure ROS in a small quantity of serum or plasma.^14^ Although previous reports describe a significant association between CKD and ROS, there is no established clinical ROS biomarker for CKD patients. In the present study, we found elevated serum DROM levels in risk factor-matched CKD patients compared with risk factor-matched non-CKD patients, and that there was no significant correlation between eGFR and serum DROM levels in CKD patients (data not shown). Similar to the present study, previous clinical studies have reported that eGFR was not associated with DROM levels in patients with low eGFR (<60 mL/min/1.73 m^2^),^15,16^ indicating that serum DROM levels were not affected by renal function in CKD patients. The current study suggests that oxidative status estimated by serum DROM levels independently increased because of a complication in CKD. The difference of DROM levels between risk factor-matched CKD patients and risk factor-matched non-CKD patients was significant but small, because DROM values in non-CKD patients (338.5 [299.8–384.3] U.CARR) were relatively high compared to normal reference levels of DROM as reported previously (250–300 U.CARR). The reasons why non-CKD patients had relatively high DROM levels in this study could be due to high prevalence of risk factors, such as age and the presence of hypertension, dyslipidemia and DM, in risk factor-matched non-CKD patients. These suggest that serum DROM levels might be increased by coexisting of above-mentioned risk factors. However, despite these effects, risk factor-matched CKD patients had higher DROM values than risk factor-matched non-CKD patients, indicating that DROM values are increased only by the presence of CKD.

Serum DROM levels generally reflect total oxidant capacity, comprising mainly lipid peroxidation-induced hydroperoxide. Vaziri et al previously reported that plasma levels of malondialdehyde, a marker of lipid peroxidation by ROS, were significantly elevated in CKD model rats,^17^ which is comparable with the present clinical study. There are many basic reports indicating the significant association between CKD and oxidative status, which is regulated by the balance between oxidant- and antioxidant systems. It is reported that nicotine adenine dinucleotide phosphate (NADPH) oxidase, one of the major sources of ROS in various cells, is activated in renal tissues,^18^ and is closely associated with the pathophysiology of CKD.^19^ A recent clinical study demonstrated that activated NADPH oxidase in renal tissues was closely involved in renal microvascular dysfunction,^20^ leading to the progression of CKD.^21^ Kim et al reported that prominent ROS in the kidney are caused by the downregulation of antioxidant enzymes such as catalase, superoxide dismutase, glutathione peroxidise, and heme oxygenase-1 in 5/6 nephrectomized CKD model rats.^22^ However, detailed molecular mechanisms behind ROS production in CKD are not fully understood, and further research to elucidate the molecular mechanisms of elevated oxidant systems and/or reduced antioxidant systems is required.

Because CAD is the main cause of death among cardiovascular events in CKD patients, risk stratification is necessary in clinical settings. Recently, we reported that the transcardiac gradient of DROM was elevated in CAD patients and that serum DROM levels were associated with future cardiovascular events in these patients.^5^ Our results in the present study also show that CKD patients with CAD had high DROM levels compared with those without CAD.

Because CKD patients are known to be predisposed to contrast-induced nephropathy, invasive diagnostic modalities such as CAG for CKD patients are relatively unfavorable. The results of the present study suggest that noninvasive measurement using the DROM test could be a useful method for cardiovascular risk assessment of CKD patients.

There are few predictive biomarkers for the occurrence of cardiovascular events in CKD patients. Conventional cardiovascular risk factors such as Framingham risk score might not reflect the prognosis of CKD patients,^23,24^ and no biomarker of ROS is established as a predictive parameter for the future occurrence of cardiovascular events in CKD patients. Walter et al reported the usefulness of a biomarker of hydroperoxide as a prognostic marker for CAD patients.^4^ In the present study, we examined the prognostic importance of DROM, reflecting mainly hydroperoxide levels, in patients with CKD. We found that DROM was a significant prognostic marker for future cardiovascular events in CKD patients, and high-DROM was as an independent and significant predictor of future cardiovascular events in CKD patients. The oxidative status as assessed by DROM measurements could provide clinically useful information for cardiovascular risk prediction in CKD patients. However, this study was observational, and there was no intervention with antioxidative drugs. The renin–angiotensin system is closely involved in the pathophysiology of CKD via overproduction of ROS,^25^ and renin–angiotensin system blockers such as ACE-I and ARB are reported to improve the prognosis of CKD.^26^ By contrast, few clinical trials have demonstrated the beneficial effects of direct antioxidant therapies not only for CKD patients but also for CAD patients. A recent clinical study, the Bardoxolone Methyl Evaluation in Patients With Chronic Kidney Disease and Type 2 Diabetes (BEACON) trial,^27^ reported that CKD patients with DM treated with a nuclear factor-erythroid 2-related factor 2 (Nrf2) activator, bardoxolone methyl, an antioxidant drug which improved eGFR levels in CKD patients,^28^ unexpectedly developed cardiovascular events, especially hospitalization for heart failure. The effects of Nrf2 activator for coronary-related events in CKD patients were not elucidated in that trial. Additionally, the BEACON trial has a few problems; for example, inappropriate methods and dosages of Nrf2 to suppress ROS production in CKD and unmatched basal cardiac functions between antioxidant- and placebo groups. Additional interventional large-scale studies are necessary to determine whether antioxidant drugs are effective at reducing the risk of cardiovascular events in CKD patients.

The present study has some limitations. First, it was a single-center design with a relatively small patient population. A large multiracial, multicenter study is required to determine the importance of DROM in CKD. Second, all enrolled patients had suspected CAD and underwent CAG to investigate whether DROM could be a useful biomarker for detecting the complication of CAD and for the risk stratification in CKD patients. Therefore, selection bias is possible as enrolled patients had more coronary risk factors than the general population, and DROM levels in CKD patients could have been influenced by such coronary risk factors. However, this study clearly demonstrated that CKD patients with CAD had higher DROM levels than those without CAD after matching risk factors.

Despite these limitations, our study provides novel evidence for the diagnostic and prognostic significance of DROM in CKD patients. Measurement of serum DROM levels might provide clinical benefits for risk stratification of CKD patients.
